# Memory T Follicular Helper CD4 T Cells

**DOI:** 10.3389/fimmu.2015.00016

**Published:** 2015-02-02

**Authors:** J. Scott Hale, Rafi Ahmed

**Affiliations:** ^1^Department of Microbiology and Immunology, Emory Vaccine Center, Emory University School of Medicine, Atlanta, GA, USA

**Keywords:** T follicular helper cells, memory T cells, Bcl6, CXCR5, helper T cells

## Abstract

T follicular helper (Tfh) cells are the subset of CD4 T helper cells that are required for generation and maintenance of germinal center reactions and the generation of long-lived humoral immunity. This specialized T helper subset provides help to cognate B cells via their expression of CD40 ligand, IL-21, IL-4, and other molecules. Tfh cells are characterized by their expression of the chemokine receptor CXCR5, expression of the transcriptional repressor Bcl6, and their capacity to migrate to the follicle and promote germinal center B cell responses. Until recently, it remained unclear whether Tfh cells differentiated into memory cells and whether they maintain Tfh commitment at the memory phase. This review will highlight several recent studies that support the idea of Tfh-committed CD4 T cells at the memory stage of the immune response. The implication of these findings is that memory Tfh cells retain their capacity to recall their Tfh-specific effector functions upon reactivation to provide help for B cell responses and play an important role in prime and boost vaccination or during recall responses to infection. The markers that are useful for distinguishing Tfh effector and memory cells, as well as the limitations of using these markers will be discussed. Tfh effector and memory generation, lineage maintenance, and plasticity relative to other T helper lineages (Th1, Th2, Th17, etc.) will also be discussed. Ongoing discoveries regarding the maintenance and lineage stability versus plasticity of memory Tfh cells will improve strategies that utilize CD4 T cell memory to modulate antibody responses during prime and boost vaccination.

## Introduction

Effective B cell responses to infectious diseases or immunization require the assistance of CD4+ helper T cells. A specialized subset of CD4 T cells named T follicular helper (Tfh) cells are required for providing this help to antigen-specific B cells. Without cognate Tfh help, activated B cells are unable to generate and maintain the germinal center response that is required for efficient somatic hypermutation of immunoglobulin genes and the selective processes that facilitate affinity maturation of antibodies (1, 2). Furthermore, the germinal center reaction is the origin of long-lived memory B cells and long-lived plasma cells that populate the periphery and bone marrow (respectively), and provide long-term antibody-mediated protection against (re)exposure to pathogens (3). Thus, Tfh cells play a critical role in the generation of effective and long-lived humoral immune responses to antigens (1).

T follicular helper cells were first identified as a subset of CD4 T cells isolated from human tonsils (4, 5). These cells expressed the B cell follicle homing chemokine receptor CXCR5 and the inducible costimulator (ICOS), and localized within the germinal center (4, 5). Furthermore, these human tonsil CXCR5+ cells (compared to CXCR5−CD45RO+ T cells) efficiently promoted production of class switched immunoglobulin (Ig)G and IgA in T cell:B cell co-culture assays (4–6). Interestingly, CXCR5+ CD4 T cells from human blood, which were presumed to be the memory counterparts of CXCR5+ cells in tonsils and lymph nodes, did not efficiently produce the Th1 signature cytokine IFNγ or the Th2 cytokines IL-4, IL-5, and IL-13 (5). Together, these studies suggested that the CXCR5+ Tfh cells represented a novel subset of helper T cells with the specific functions of providing help for B cell responses and that are distinct from Th1 and Th2 cells. Since these initial seminal reports describing Tfh cells, extensive studies have demonstrated that while Tfh cells share certain similarities with Th1, Th2, and Th17 cells (depending upon context of infection or vaccination), these cells have unique developmental requirements and distinct phenotypic, homing, and functional qualities compared to other T helper cell lineages (Th1, Th2, Th17, Treg) (1).

**Figure 1:**
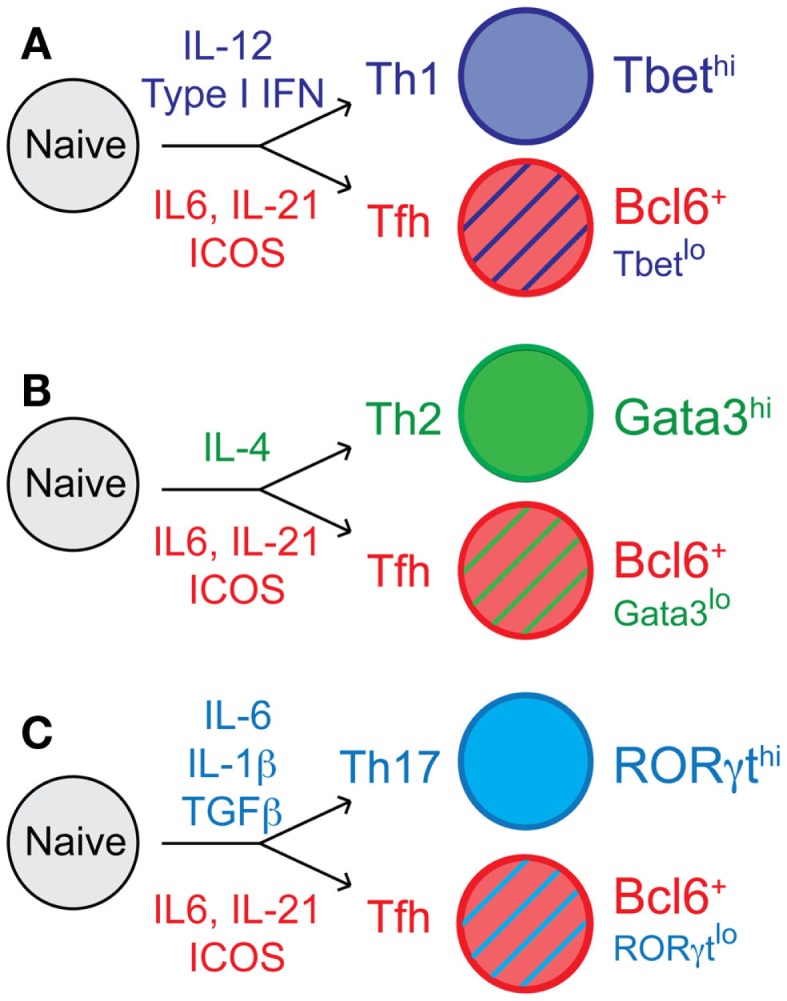
**T follicular helper cell differentiation and context-dependent Tfh cell heterogeneity**. Following activation of naïve CD4 T cells, cells proliferate and undergo fate decisions in response to cytokines and other differentiating factors. Tfh cell differentiation is influenced by IL-6 and IL-21, and dependent upon ICOS signaling for expression of the transcription factor Bcl6. Cytokines including IL-12, IL-4, IL-1β, and many others, direct **(A)** Th1, **(B)** Th2, and **(C)** Th17 cell differentiation, respectively. The context-dependent cytokine milieu also influences Tfh cell differentiation; thus, Tfh effector cells can express some low/intermediate levels of the transcription factors Tbet, Gata3, and RORγt, which are associated with the **(A)** Th1, **(B)** Th2, and **(C)** Th17 cell lineages, respectively.

Upon activation with cognate antigen by dendritic cells, antigen-specific CD4 T cells can differentiate to become various types of effector CD4 T cells with specific roles in promoting anti-pathogen immune responses (Figure 1). Early differentiation toward the Tfh lineage requires ICOS expression and signaling to induce expression of the transcriptional repressor Bcl-6 (7). Bcl6 is required for Tfh cell generation, maintenance, and function, establishing Bcl6 as a central regulator in Tfh cell lineage development (Figure 1) (8–10). Bcl6 expression promotes Tfh differentiation, at least in part by suppressing transcription of the transcriptional regulators Tbet, RORγt, GATA3, and Blimp-1 (8–10), and through other potential mechanisms, such as the repression of microRNAs (10). In addition, cytokines such as IL-6 and IL-21 (11), and other molecules such as SAP are important for Tfh differentiation and function (12, 13). Importantly, interactions with cognate B cells were required for amplifying the expression of Bcl6 for the maintenance of the Tfh phenotype during the immune response (7). During Tfh differentiation, Bcl6 plays an important role in suppressing Blimp1 (8), which is a regulator of Th1, Th17, and Th2 lineage cells (1). Thus, the promotion of Bcl6 coupled to the repression of Blimp1 plays a role in the differentiation, function, and possibly the stability of Tfh cells in relation to other T helper cell subsets. Additional transcription factors such as Maf (14, 15), Ascl2 (16), and others play important roles in Tfh cell differentiation and/or function (1).

T follicular helper cells have developmental requirements that differ from those that promote Th1, Th2, and Th17 effector cell development (1). However, depending upon the type of infection (viral, helminth, fungal, etc.) or immunization and the inflammatory environment that is generated, Tfh cells can express low to intermediate levels of Tbet, Gata3, or RORγt (Figure 1) (17–20). This intriguing context-dependent transcription factor expression in Tfh cells results in a variety of Tfh cell subsets that can express low levels of specific cytokines that can influence antibody class switching (17, 21, 22). Thus, studying the complex relationship between Tfh cells and their non-Tfh cell counterparts, and whether they remain phenotypically and functionally distinct throughout the immune response and beyond is critical to understanding Tfh cell commitment and flexibility and to determining their biologically important roles during specific types of immune responses.

## Lineage-Committed Memory T Helper Cells

The adaptive immune system responds to infectious challenge with two major goals. The first goal is to generate sufficient numbers of antigen-specific effector cells to limit and clear the pathogen. The second priority is to provide long-lasting immunity that will defend the host from subsequent exposure to the pathogen (23). The activation-driven proliferation and lineage differentiation of CD4 T cells *in vivo* is accompanied by the progression of memory differentiation. Following clearance of antigen, the majority (approximately 90–95%) of antigen-specific effector T cells undergo apoptosis, leaving behind a population of memory cells. In some experimental models, antigen-specific CD4 memory T cells gradually decline over long periods of time (24, 25). For example, *Listeria monocytogenes* infection-induced memory CD4 T cells are present at relatively high frequencies 90 days post-infection; however, by approximately 250 days post-infection, the population has largely disappeared from the spleen and lymph nodes (25). In contrast, human studies reveal that long-lived vaccinia-specific memory CD4 T cells are relatively stable for at least several decades after smallpox vaccination (26, 27).

Memory T cells possess many important features compared to their naïve CD4 T cell precursors. First, antigen-specific memory cells are found in increased numbers relative to their naïve antigen-specific precursors, providing better coverage and a more rapid cellular response upon pathogen rechallenge. Second, memory cells are not restricted to blood circulation and secondary lymphoid organs, but instead may also traffic to and reside in non-lymphoid tissues, where they may rapidly exert effector functions if their specified pathogen gains entry to that particular anatomical site. Third, memory T cells have undergone changes in cell-intrinsic programing, allowing them to rapidly recall their effector functions, such as prompt expression of specific effector cytokines, chemokines, and cytotoxic molecules. Finally, memory cells are long-lived, and a central feature of their longevity is dependent on their ability to undergo homeostatic proliferation in the absence of antigen (23, 28).

Combining the study of T helper lineage differentiation and T cell memory differentiation *in vivo* following vaccination or infection is incredibly complex. However, it provides the opportunity to gain vital understanding into the heterogeneity and lineage commitment and flexibility of the resulting antigen-specific memory CD4 T cells that will be informative for ongoing and future vaccine discovery/development efforts. It has become clear that among the vast heterogeneity of memory CD4 T cells, many memory cells demonstrate commitment to a previously defined T helper lineage. The existence of Th1-commited long-lived memory CD4 T cells was demonstrated in BAC transgenic mice that used a reporter to indicate transcription of the *Ifng* gene. In this study, Harrington et al. demonstrated that these memory cells were derived from the effector Th1 cells, and rapidly recalled IFNγ expression at the effector phase (29). Several other studies similarly found that subsets of LCMV-specific and *Listeria*-specific memory CD4 T cells with distinct phenotypes were committed Th1 memory cells that recalled IFNγ expression and expression of other Th1 effector molecules (20, 30, 31). One study utilized an IL4 IRES EGFP reporter mouse to demonstrate that Th2 effector cells (EGFP+) generated from *N. brasiliensis* infection could provide anti-parasite protective immunity after adoptive transfer into immunocompromised recipient mice and 30 days resting before parasite challenge (32). Similarly, *Trichuris*-specific Th2 memory cells recall their Th2 effector functions and mediate anti-parasite immunity (33). While Th17 CD4 T cells generated by intranasal *Listeria* infection (a Th1 pathogen) do not form memory cells (25), *Candida* and other fungal vaccines, as well as other conditions have been shown to induce Th17 memory cells *in vivo* (34–36). Together, these studies demonstrate the characteristics and programs of polarized effector Th1, Th2, and Th17 cells that are generated early during effector differentiation are preserved in resting memory cells. Importantly, these effector programs are recalled after reactivation *in vivo* to infectious challenge in an antigen-specific manner, and with the appropriate T helper effector response to effectively eliminate the pathogen.

## T Follicular Helper Memory Cells

The establishment of Tfh cells as an independent effector T helper subset, and the factors that drive Tfh differentiation being defined, provides a strong rationale for exploring whether Tfh cells that progress to become memory cells maintain their Tfh attributes following resolution to the immune response. However, given the potential flexibility/plasticity of Tfh cells toward repolarization (37), one might predict that Tfh cells generate non-committed memory CD4 T cells. Several fundamental questions exist regarding the relationship of Tfh cells and memory cells. First, do Tfh cells survive to become memory cells? Second, do memory cells derived from Tfh cells maintain their commitment/programing to recall Tfh effector cells, or, instead possess pluripotency/plasticity to become cells of other T helper lineages? The answer to these questions provides profound insight into the importance of how Tfh differentiation during primary immune responses to natural infection and vaccination have the potential to influence secondary antibody responses. Several recent studies have provided insight into the existence and characteristics of memory Tfh cells and their capacity to recall Tfh-specific effector functions following reactivation with antigen.

One early study investigating memory Tfh cells in mice described a population of CXCR5+ICOS+ cells in the draining lymph nodes 30–56 days following immunization with pigeon cytochrome C in adjuvant. Compared to day 7 Tfh effector cells, day 30 PCC-specific cells had decreased ICOS and OX40 expression. The authors reported that these were a subset of memory Tfh cells with enhanced recall capacity upon immunization 6–8 weeks after priming (38). However, this study further reported the persistence of antigen for more than 75 days after immunization and the maintenance of CD69 on these PCC-specific CD4 Tfh cells indicate that this experimental system promotes long-lived antigen depots. Thus, the CXCR5+ICOS^lo^ cells identified in this study cannot be clearly distinguished as true memory cells that survive in the absence of antigen (38).

A study by MacLeod and Marrack provided a stronger basis for the existence of memory CD4 T cells with accelerated Tfh function during recall responses (39). Their study demonstrated that on a per cell basis, antigen-specific memory CD4 T cells (compared to Ag-specific naïve cells) provided accelerated B cell responses and antibody class switching. Interestingly, this accelerated B cell helper capacity was contained within the CXCR5+ subset of memory CD4 T cells, resulting in higher OVA-specific IgG1 titers following adoptive cell transfer and followed by immunization. The authors suggested that CXCR5 chemokine receptor expression promotes their more rapid migration of reactivated memory CD4 T cells to B cell follicles, allowing them to provide accelerated help to the B cell response (39).

It was unknown whether Tfh cells were able to survive and become memory cells. To address this question, Weber et al. used protein immunization in CFA to induce TCR transgenic CD4 effector T cells, sorted them into CXCR5− and CXCR5+ subsets, and then adoptively transferred these into naïve recipient mice (40). Fourteen days later, adoptively transferred CXCR5+ effector cells had become CXCR5 low/negative. After resting these cells in naïve recipient mice for several weeks, a large proportion of transferred Tfh cells quickly recalled a Tfh phenotype within 2.5 days following re-immunization, expressing higher levels of PD-1, Bcl6, CXCR5, and IL-21 compared to non-Tfh cells and primary effector cells (40). While the time periods that these cells were rested in the absence of antigen were relatively brief, this study was highly suggestive that effector Tfh cells could be maintained in the absence of antigen and preferentially recall a Tfh phenotype. A study by Choi and colleagues addressing the fate commitment of Tfh cells demonstrated that day 3 LCMV-specific Tfh effector cells, following adoptive transfer into day 3 LCMV-infected (infection-matched) recipient mice, were maintained up to 45 days later and retained CXCR5 surface expression (41). An additional study demonstrated that CXCR5+RFP+ (from Bcl6 RFP reporter mice) OTII cells induced by Ova/CFA immunization can persist following adoptive transfer for 20 days, and upon OVA+CFA immunization preferentially recall Tfh cells in the draining lymph node (42). Together, these studies clearly demonstrate that not all Tfh effector cells are terminally differentiated and fated to die. Instead, some Tfh effector cells progress to become memory cells that have some similar features compared to their Tfh effector predecessors.

Our study investigated the differentiation of Th1 and Tfh cells following acute LCMV infection using the adoptive transfer and analysis of LCMV-specific CD4 T cells (SMARTA TCR transgenic) induced by acute viral infection (20). Following the clearance of acute LCMV infection and the corresponding contraction of virus-specific CD4 T cells, both Th1 and Tfh memory subsets are maintained at relatively stable numbers for approximately 60–150 days post-infection (20). Resting PD-1− and ICOS−CXCR5+Ly6c^lo^ memory CD4 T cells shared phenotypic and gene expression similarities to CXCR5+Ly6c^lo^ effector Tfh cells, suggesting a direct lineage-relationship between these populations at the different stages of the immune response. Adoptive transfer of CXCR5+ memory subsets followed by LCMV Armstrong challenge resulted in Tfh secondary effector cells with ability to rapidly recall a Tfh effector phenotype and promote the generation of germinal center B cells. In addition, when CXCR5+ memory cells were transferred to B cell deficient recipient mice that were then infected, a large proportion of the resulting effector cells recalled and sustained a Tfh-like phenotype, while primary effector cells generated from naïve (uncommitted) SMARTA cells did not. These latter results indicate that CXCR5+ memory cells have acquired and maintained a Tfh-biased cell program relative to their naïve cell counterparts (20). Interestingly, while there was some apparent flexibility by some CXCR5+ memory cells to generate CXCR5− Th1-like secondary effector cells, cell-intrinsic restrictions impaired the Th1 effector program, resulting in poor granzyme B and IFNγ expression. The results from this study argue in favor of Tfh lineage commitment within the CXCR5+ Tfh memory cell population (20).

In contrast to these many studies that report the existence of memory Tfh cells that promptly and preferentially recall Tfh phenotype and function upon rechallenge, one study reported that *L. monocytogenes* specific (identified by MHC class II tetramer) CXCR5+ memory CD4 T cells are pluripotent, promoting the recall of both Tfh cells and non-Tfh (Th1) cells (31). Another study of influenza infection in IL-21 GFP reporter mice showed that the adoptive transfer of polyclonal CXCR5+GFP+ Tfh CD4 effector cells gave rise to memory cells with sufficient plasticity to generate secondary effectors of both Tfh cells and non-Tfh cells. Thus, Luthje et al. conclude that Tfh effector cells are “uncommitted” regarding their T helper lineage (43). Interestingly, these studies both used adoptive transfer of polyclonal CD4 T cells (31, 43), while studies demonstrating relative Tfh commitment of CXCR5+ Tfh memory cells utilized TCR transgenic cells specific for a single epitope (20, 40, 42). Because strength and/or longevity of TCR signaling influences Th1 versus Tfh differentiation (44, 45) as well as memory CD4 T cell differentiation (46), individual TCRs within the Tfh effector population may confer varying degrees of lineage commitment in CXCR5+ Tfh memory cells. Going forward, it will be interesting to determine whether different TCRs, infection and immunization systems, and other factors play a role in the degree of plasticity versus commitment in memory Tfh cells.

**Figure 2:**
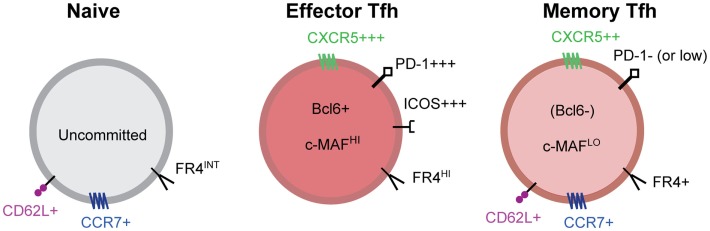
**Markers of effector and memory Tfh cells**. Tfh effector CD4 T cells express high levels of the chemokine receptor CXCR5, the inhibitory receptor PD-1, ICOS, the transcriptional repressor Bcl6, the transcription factor c-Maf, and many other molecules. After antigen clearance, resting memory Tfh cells no longer express Bcl6, ICOS, and IL-21 and many other Tfh associated molecules. Tfh memory cells are characterized by intermediate expression of CXCR5 and other Tfh related molecules and the absence of activation dependent molecules. Bcl6 expression is not detected in memory Tfh cells; however, Maf and other transcription factors are maintained at low/intermediate levels. PD-1 is absent from antigen-specific memory Tfh cells in mice, however, human CXCR5+CXCR3− memory Tfh cells maintain low levels of PD-1 expression that is not dependent on TCR signaling. Folate receptor 4 (FR4), a molecule that is highly expressed on effector Tfh cells, is maintained on CXCR5+ memory Tfh cells.

There are many well described Tfh cell markers; however, there are relatively few markers useful for clearly distinguishing resting memory Tfh cells. Antigen-specific memory Tfh cells express CXCR5, but lack Bcl6, ICOS, and expression of many other Tfh molecules (20, 41, 47) (Figure 2). In addition, memory Tfh cells do not express CD69 (20), an indicator of ongoing antigen stimulation. A recent study demonstrated that folate receptor 4 (FR4), a surface receptor that is upregulated on Tfh cells but not Th1 cells during acute viral infection, is maintained on the surface of CXCR5+ memory Tfh cells (48). To date, the best marker for identifying memory Tfh cells is CXCR5 (18, 20, 41, 47) (Figure 2); however, not all CXCR5+ memory cell subsets exhibit specific Tfh function upon reactivation (47). Figure 2 shows some of the phenotypic markers associated with the memory Tfh cell phenotype.

**Figure 3:**
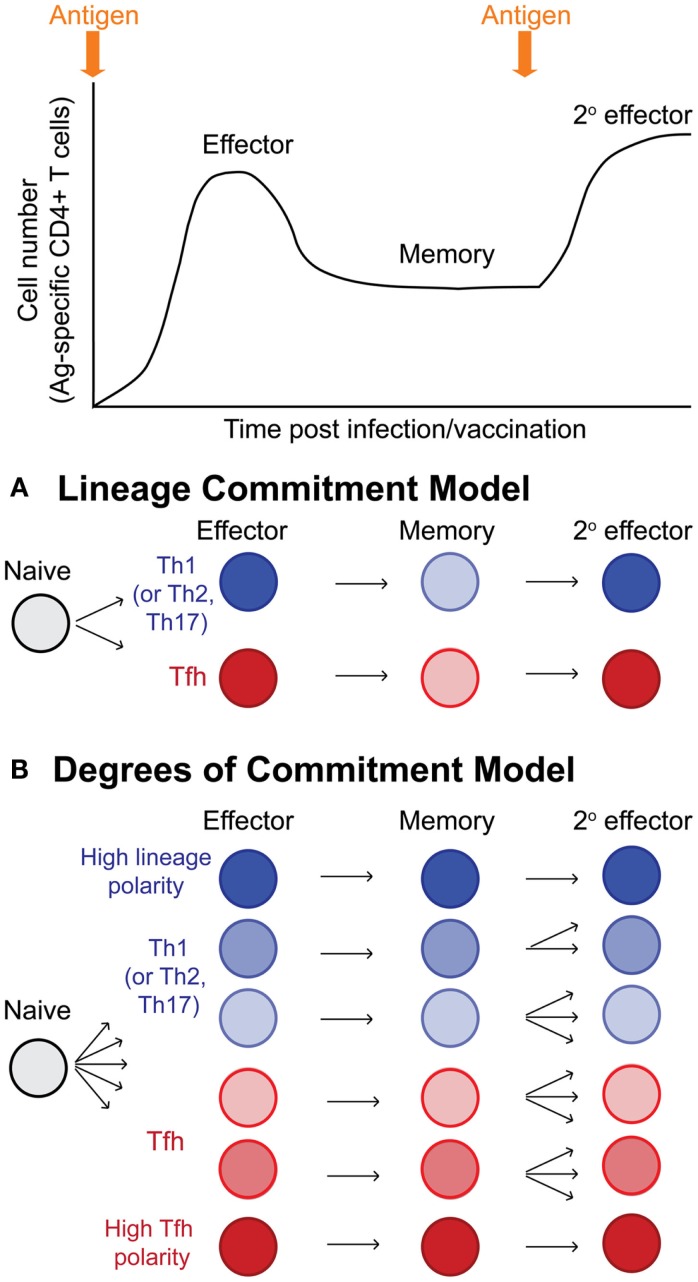
**Models of T helper lineage commitment in memory CD4 T cells**. Following activation, naïve CD4 T cells undergo dramatic antigen dependent proliferation to generate effector CD4 T cells. Following antigen clearance, the majority of effector cells undergo apoptosis, leaving behind a pool of antigen-specific memory cells. Upon reinfection or antigen boost, memory cells become reactivated and proliferate, generating secondary effector cells. **(A)** Potential model for lineage commitment of Tfh memory cells. In this model, antigen-specific CD4 T cells differentiate to become either lineage-committed Tfh cells or lineage-committed effector cells of other lineages (either Th1, Th2, or Th17 depending upon the context of infection or inflammatory stimulus). Resting memory Tfh and non-Tfh cells would maintain their lineage-specific programing, and upon reactivation, would faithfully recall their previously defined T helper lineage phenotype and functions. **(B)** In this second model, antigen-specific CD4 T cells differentiate toward either Tfh or non-Tfh (Th1, Th2, or Th17) effector cells; however, depending upon the integration of many different stimuli (TCR signals, inflammation, interactions with various antigen-presenting cells, etc.), individual effector cells become transcriptional and epigenetically programed with varying degrees of polarity toward Tfh or non-Tfh cell lineages. Following antigen clearance and T cell contraction, the antigen-specific memory pool is highly heterogenous, being comprised of cells with varying degrees of lineage commitment and T helper lineage-specific recall potential, including highly committed Tfh memory cells, more plastic/pluripotent Tfh and non-Tfh (Th1, Th2, and Th17) memory cells, and highly committed non-Tfh (Th1, Th2, and Th17) memory cells.

It has become clear that Tfh effector cells can become memory Tfh cells with cell-intrinsic programs that promote the recall of Tfh cells upon reactivation. It is therefore possible to propose models of T helper cell differentiation wherein naïve CD4 T cells give rise to Tfh and other T effector lineage cells (Th1, Th2, or Th17 depending upon context of the pathogen or inflammation), and these effector cells give rise to memory cells that strictly maintain their lineage commitment and recall their lineage upon reinfection or boosting with antigen (Figure 3A). However, memory T cell populations are characterized by a vast degree of phenotypic and functional heterogeneity. A second and more comprehensive model would suggest that as effector cells differentiate, individual cells acquire varying degrees of programing toward the Tfh or other T effector lineage cells that can be stably maintained in resting memory cells (likely through epigenetic mechanisms) (Figure 3B). The resulting population of antigen-specific memory cells would then contain a wide spectrum of cells with varying degrees of lineage commitment versus pluripotency/plasticity (Figure 3B). This latter model may account for why some studies report lineage commitment within Tfh cell subsets, while others studies report relative non-commitment or plasticity. Future mechanistic studies will provide improved insight into the heterogeneity of memory CD4 T cells in relation to their T helper lineage commitment and recall potential. Furthermore, determining whether Tfh memory cells are homeostatically maintained over time at very late memory timepoints remains to be explored.

## Human T Follicular Helper Memory Cells

The original reports describing CXCR5+ Tfh cells in human tonsils had differing ideas regarding the existence of memory Tfh cells. One report described that tetanus-antigen-specific proliferation was induced in CD4+CD45RO+CXCR5−but not CD4+CD45RO+CXCR5+ cells, thus suggesting that Tfh cells likely disappear along with germinal centers, and are prone to apoptosis due to their high levels of Fas expression (4). In contrast, the other report showed that unlike the activated tonsillar CXCR5+ CD4 T cells that were CD69+HLA-DR+ICOS+, circulating CXCR5+ cells in human blood are CD69−HLA-DR−ICOS−, and hypothesized that these blood CXCR5+ cells represent memory Tfh cells (5). Since these initial observations, whether activated human Tfh cells become memory T cells, and whether they maintain their Tfh function upon reactivation has remained unclear. Recent reports have shed light on the nature of human CXCR5+ memory Tfh cell ontogeny and function (18, 37, 47).

One study described a population of circulating human CXCR5+ central memory CD4 T cells that expressed CXCL13 and promoted B cells to undergo plasma cell differentiation and Ig secretion (37). Another recent study investigating circulating CXCR5+ CD4 T cells in human blood revealed that these cells share functional characteristics with Tfh cells. CXCR5+ cells promoted the isotype switching and antibody production of IgG, IgA, and IgE isotype switched antibodies in T:B cell co-culture experiments, while CXCR5− cells did not promote switched antibody production (18). Their study further categorized the CXCR5+ population into CXCR3+CCR6− (Th1-like), CXCR3−CCR6+ (TH17-like), and CXCR3−CCR6− (Th2-like) subsets. While CXCR5+CXCR3+CCR6−cells did not provide help in these co-culture assays, CXCR5+CXCR3−CCR6+ cells promoted high levels of IgG and IgA antibodies, while CXCR5+CXCR3−CCR6−cells promoted high IgG and IgE, and intermediate levels of IgA. This study highlights the function of reactivated CXCR5+ CD4 T cells from peripheral blood to promote antibody production, and further defines the vast heterogeneity of CXCR5+ cells in relation to the Th1, Th2, and Th17 lineage of cells (18). The CD69− and ICOS− phenotype of these circulating CXCR5+ cells (prior to reactivation) suggests that they are resting memory cells (18). However, inability to identify antigen-specific cells and relate them to a known time of antigen exposure lead to difficulty in concluding whether they are indeed true memory Tfh cells that persist long-term in the absence of antigen and maintain their lineage characteristics in their resting state.

Locci et al. profoundly advanced the understanding of human Tfh memory cells and clarified their existence. Their study identified that blood CXCR5+CXCR3− cells that had a non-activated/resting phenotype could be stratified into either PD-1− or PD-1^low^ subsets (47). Interestingly, low PD-1 expression was stably maintained on this latter subset of cells during 20 day culture *in vitro* in the absence of TCR stimulation, suggesting that sorted CXCR5+PD-1^low^ cells express these molecules (PD-1 and CXCR5) as a part of the Tfh transcriptional program. Indeed, the transcriptional profile of these CXCR5+CXCR3−PD-1^low^ cells is similar (albeit reduced) to that of germinal center Tfh cells from human tonsils, and these cells promoted the highest IgG antibody production and plasmablast differentiation in T:B cell co-culture experiments compared to other subsets of CXCR5+ cells (47). Furthermore, characterization of tetanus-specific CD4 T cells in healthy human donors (identified using HLA tetramers bearing tetanus peptide), demonstrated the existence of this CXCR5+CXCR3−PD-1^low^ phenotype among resting antigen-specific memory cells (Figure 2). Together, these experiments define the phenotype and function of human memory Tfh cells and combine to fortify the idea that memory Tfh cells exist within the pool of resting human antigen-specific memory cells (47).

Defining strategies and markers to distinguish and characterize antigen-specific human Tfh effector and memory subsets is difficult, and is the focus of many recent and current lines of research (18, 47, 49–51). The vast heterogeneity of effector and memory CD4 T cells in human blood and other tissues has led to difficulty in understanding what Tfh-like cell subsets serve as the best correlates of effective antibody responses, and what subsets actually provide the help for the B cell response *in vivo* (51). Some of the differences for Tfh and/or Tfh memory markers utilized in these various studies may result from the different types of infections or vaccinations studied, the timepoints analyzed (early after vaccination versus long-lived memory cells), and the varying analysis of “bulk” memory populations of unknown antigen-specificities versus analysis of tetramer+ pathogen specific memory cells (18, 47, 49–51). As better markers, reagents, and methods for studying antigen-specific Tfh responses become available, the developmental requirements and heterogeneity of human memory Tfh cells and their relationship to other T helper cell subsets will be better understood. This will open doors to more clearly define the specific roles that Tfh memory cells play during recall responses by human Tfh cells during boosting or in response to natural infection.

## Preserving the Tfh Program in Resting Memory Tfh Cells

**Figure 4:**
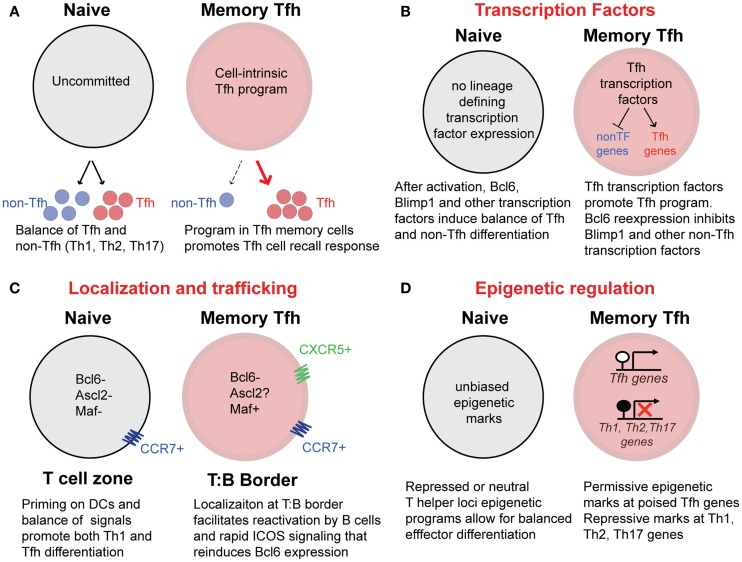
**Potential mechanisms that promote Tfh recall responses from memory Tfh cells**. **(A)** Differentiated CXCR5+ memory Tfh cells possess cell-intrinsic programs that promote the preferential generation of Tfh secondary effectors cells upon reactivation with antigen. In contrast, uncommitted naïve CD4 T cells are pluripotent, with the capacity to generate a balance of Tfh and non-Tfh (Th1, Th2, Th17) effector cells following activation. **(B)** The maintenance of Tfh associated transcription factors may promote the recall of the Tfh cell program and inhibit the differentiation of other T helper lineages following activation. The transcription factor Maf, is maintained at low levels in human Tfh memory cells. While Bcl6 expression is not detectable in memory Tfh cells, it is possible that soon after reactivation, rapid reexpression of Bcl6 directs Tfh gene expression as well as inhibits Blimp1 and high expression of Tbet, Gata3, and RORγt. **(C)** CXCR5 and CCR7 coexpression on memory Tfh cells may provide localization of memory Tfh cells along the T:B cell border either before or early after reactivation, promoting increased interactions with B cells that can reinforce the Tfh cell phenotype and function. In contrast, CCR7+ (CXCR5−) naïve CD4 T cells will become activated in T cells zones by antigen-bearing dendritic cells. **(D)** Epigenetic programs acquired during Tfh effector differentiation can be maintained in resting memory Tfh cells throughout homeostatic proliferation. Transcriptionally permissive histone modifications and unmethylated DNA at “poised” Tfh associated loci in memory Tfh cells will promote the rapid reexpression of these genes upon reactivation. In addition, repressive histone modifications and DNA methylation will prevent reexpression of Th1, Th2, and Th17-associated genes.

A complex combination of cytokines, transcription factors (52), STAT molecules (53), and epigenetic changes (54, 55) combine to delineate both the initial differentiation and stability of T helper lineages. Extrinsic factors that drive CD4 T cell activation and T helper cell differentiation are absent after antigen/pathogen clearance. Consequently, transcription factors such as Tbet and Bcl6 and other molecules that define T helper lineages are downregulated on memory CD4 T cells (20, 31, 40, 41). A critical question regarding the existence of CXCR5+ Tfh memory cells (and other specialized T helper cell memory subsets) remains: how do memory cells maintain lineage commitment and remember the gene expression programs of their previously defined T helper lineage in the absence of antigen and inflammatory signals (Figure 4A) (56, 57)? While the answer to this question for memory Tfh cells is currently not resolved, it is likely that the reexpression of key transcription factors, as well as the epigenetic state of genes related to Tfh cell development and function, play an important role in directing Tfh memory cells toward recalling a secondary Tfh response. Potential mechanisms (that are not mutually exclusive – and more likely work in concert) for preserving the program of Tfh memory cells that promote the recall of Tfh secondary effector cells are shown in Figures 4B–D.

Bcl6 plays a critical role in defining the phenotype and function of Tfh cells (8–10). Although all of the mechanisms by which this transcriptional repressor positively directs the Tfh gene expression program, Bcl6 expression can induce CXCR5 expression (9, 15), likely through an indirect mechanism. Bcl6 overexpression in naïve human CD4 T cells promotes the regulation of genes required for germinal center trafficking and interactions with B cells, and in cooperation with Maf induces expression of other key Tfh genes (15). It is clear that while many Tfh associated genes are downregulated in resting CXCR5+ memory cells, these cells maintain a Tfh-like gene expression profile for many other Tfh associated genes (20, 47). Bcl6 transcript was not detectable in memory Tfh cells (20, 41, 42, 47). However, this does not rule out that very low levels of Bcl6 expression could bias the recall of memory Tfh cells early after cell reactivation by promoting the Tfh phenotype and inhibiting Blimp1 activity (Figure 4B). Furthermore, because memory B cells can present antigen to and rapidly induce Bcl6 expression in memory Tfh cells (58), the Bcl6-dependent Tfh transcriptional program may be robustly reinforced in memory Tfh cells upon reactivation *in vivo*.

Maf gene and protein expression were found at low levels in human blood Tfh memory cells (CXCR5+CXCR3−PD-1+) (47), suggesting that this important transcriptional regulator of Tfh cell functions (14, 15) may play a role in preserving aspects of the Tfh phenotype and program in these memory cells. In addition, Ascl2, a recently described transcription factor was shown to be required for early Tfh differentiation and function (16). Overexpression of Ascl2 results in CXCR5 expression, CCR7 downregulation, and subsequent migration to the follicles. Ascl2 binds to conserved non-coding sequence regions of the *Cxcr5* locus and promotes *Cxcr5* gene expression. Interestingly, Ascl2 did not induce Bcl6 expression. It is therefore possible that Ascl2 expression in Tfh memory cells may be important for maintenance of CXCR5 expression in resting memory Tfh cells (16). Currently, it is unclear what specific roles Bcl6, Maf, Ascl2, or other transcription factors play in preserving CXCR5 expression on memory Tfh cells, and promoting maintenance of the Tfh gene expression program (Figure 4B). Upon reactivation, it is possible that CXCR5+ Tfh memory cells are already localized (or rapidly relocalize) to the T:B border, resulting in interactions with B cells that would then reinforce Bcl6 reexpression and other Tfh transcription factors (Figure 4C). In turn, this might preferentially promote the Tfh program (over other T helper lineage programs) during the secondary response (Figure 4C). Currently, it remains unclear how these and other transcription factors influence the maintenance of the Tfh program and the repression of other T helper cell lineages in Tfh memory cells.

Transcription factors that have been termed lineage “master regulators,” such as Tbet, RORγt, and Gata3 are not always limited in their expression to a single subset of differentiated CD4 T cells and can be expressed in Tfh effector cells (Figure 1) (1). The expression of these factors in Tfh effector cells is believed to be important for promoting expression of key cytokines that direct specific isotype class switching in cognate germinal center B cells (17, 21, 22). The balance of Bcl6 and Tbet has been shown to have important effects of differentiation and gene expression by Th1 and Tfh cells (59–61). Interestingly, following LCMV infection, mouse Tfh cells co-express Bcl6 and intermediate levels of Tbet (20). The resulting Tfh memory cells do not have detectable Bcl6, but surprisingly, maintain low levels of Tbet gene and protein expression (20). Despite this Tbet expression (and lack of Bcl6), adoptively transferred Tfh memory cells predominantly generate Tfh secondary effector cells following reinfection (20). This finding invokes the idea that beyond the simple balance of lineage-associated transcription factors, transcriptional programing through epigenetic modifications likely plays a role in promoting the reexpression of Tfh genes and repressing the gene expression programs of other T helper cell lineages (57).

The maintenance of gene expression and lineage-differentiation programs in the absence of inflammatory/differentiating signals is reinforced by epigenetic programs that are acquired during the initial effector T cell differentiation (54, 57). The combination of epigenetic modifications to histones to regulate the structure of chromatin and the methylation of DNA at CpG motifs that determine the binding of inhibitory methyl-binding domain proteins can be conserved in daughter cells throughout cell division (62), providing a mechanism for gene expression programs to be maintained during T cell homeostatic proliferation (57). Furthermore, transcriptionally permissive epigenetic marks are positively associated at loci of lineage-specific cytokines for each T helper lineage, while repressive marks at specific loci relevant to other T helper lineages serve to prevent inappropriate gene expression and suppress the differentiation toward alternative lineages (54, 57). Lu et al. observed that positive chromatin marks were associated in Tfh-like cells at the *Tbx21*, *Gata3*, and *Rorc* loci, and that these cells could be repolarized toward Th1, Th2, and Th17 cells when restimulated under polarizing conditions (63). This study highlights that certain features of the Tfh program may partially overlap with Th1, Th2, and Th17 cell programs, possibly allowing greater plasticity toward the capacity to produce multiple lineage-defining cytokines. In addition, *in vitro* differentiated Th1 and Th2 cells (but not Th17 cells) can become Tfh cells following adoptive transfer and immunization with protein in adjuvant (42). Together, these studies demonstrate that there is a degree of flexibility between effector Tfh cells and other T helper cells. Further work is required to determine whether and how such flexibility applies to CXCR5+ memory Tfh cells generated *in vivo*. It is possible that context-dependent selective processes during memory T cell development promote antigen-specific memory T cells that are either more or less committed to Tfh differentiation compared to their effector (precursor) counterparts (Figure 3).

Methylation of CpG motifs in regulatory regions of genomic DNA serve as a transcriptionally repressive mark through recruitment of methyl-binding domain proteins (62, 64). Loss of DNA methyltransferase activity has dramatic effects upon CD4 differentiation, resulting in loss of restriction of key lineage-associated cytokine and transcription factor genes (65–69). DNA bisulfite sequencing analysis of LCMV-induced CD4 T cells revealed that the *Gzmb* locus (encoding granzyme B) became demethylated in Th1 but not Tfh effector cells. Furthermore, the maintenance of DNA methylation at the *Gzmb* locus in CXCR5+ memory Tfh cells was predictive of both their capacity to recall the Tfh effector phenotype upon reactivation with antigen *in vivo*, and also their inability to efficiently express granzyme B, even among the small fraction of Th1-like secondary effector cells that had downregulated CXCR5 expression (20). Thus, repression of Th1-associated gene expression programs (such as *Gzmb*) in memory Tfh cells likely plays an important role in the maintenance of the Tfh program in resting CXCR5+ Tfh memory cells by repressing the expression of genes associated with Th1 effector function (Figure 4D). Future work is needed to characterize permissive versus repressive epigenetic marks (for loci of lineage-associated cytokines, effector molecules, and transcription factors) in antigen-specific memory Tfh, Th1, Th2, and Th17 cells, providing needed insight into the plasticity versus commitment of memory CD4 T cell subsets induced by infection and immunization.

## Concluding Remarks

The discovery that phenotypically unique subsets of CXCR5+ memory CD4 T cells have recall potential specific for Tfh function invites important questions for future study that will inform vaccination strategies for infectious diseases. Understanding the mechanisms of how Tfh memory cells acquire and preserve the Tfh gene expression programs and preferentially recall these programs upon reactivation will provide important insight into the lineage maintenance and plasticity of these cells. Finally, future work is needed to determine the optimal ways to utilize Tfh memory cells during prime and boost immunization to promote ways that improve protective and long-lived antibody responses.

## Conflict of Interest Statement

The authors declare that the research was conducted in the absence of any commercial or financial relationships that could be construed as a potential conflict of interest.
